# Fatty Acid Esters of Phloridzin Induce Apoptosis of Human Liver Cancer Cells through Altered Gene Expression

**DOI:** 10.1371/journal.pone.0107149

**Published:** 2014-09-17

**Authors:** Sandhya V. G. Nair, Ziaullah, H. P. Vasantha Rupasinghe

**Affiliations:** Department of Environmental Sciences, Faculty of Agriculture, Dalhousie University, Truro, Nova Scotia, Canada; University of Illinois, United States of America

## Abstract

Phloridzin (phlorizin or phloretin 2′-*O*-glucoside) is known for blocking intestinal glucose absorption. We have investigated the anticarcinogenic effect of phloridzin and its novel derivatives using human cancer cell lines. We have synthesised novel acylated derivatives of phloridzin with six different long chain fatty acids by regioselective enzymatic acylation using *Candida Antarctica* lipase B. The antiproliferative effects of the new compounds were investigated in comparison with the parent compounds, phloridzin, aglycone phloretin, the six free fatty acids and chemotherapeutic drugs (sorafenib, doxorubicin and daunorubicin) using human hepatocellular carcinoma HepG2 cells, human breast adenocarcinoma MDA-MB-231 cells and acute monocytic leukemia THP-1 cells along with normal human and rat hepatocytes. The fatty acid esters of phloridzin inhibited significantly the growth of the two carcinoma and leukemia cells while similar treatment doses were not toxic to normal human or rat hepatocytes. The antiproliferative potency of fatty esters of phloridzin was comparable to the potency of the chemotherapeutic drugs. The fatty acid esters of phloridzin inhibited DNA topoisomerases IIα activity that might induce G_0_/G_1_ phase arrest, induced apoptosis via activation of caspase-3, and decreased ATP level and mitochondrial membrane potential in HepG2 cells. Based on the high selectivity on cancer cells, decosahexaenoic acid (DHA) ester of phloridzin was selected for gene expression analysis using RT^2^PCR human cancer drug target array. Antiproliferative effect of DHA ester of phloridzin could be related to the down regulation of anti-apoptotic gene (BCL2), growth factor receptors (EBFR family, IGF1R/IGF2, PDGFR) and its downstream signalling partners (PI3k/AKT/mTOR, Ras/Raf/MAPK), cell cycle machinery (CDKs, TERT, TOP2A, TOP2B) as well as epigenetics regulators (HDACs). These results suggest that fatty esters of phloridzin have potential chemotherapeutic effects mediated through the attenuated expression of several key proteins involved in cell cycle regulation, DNA topoisomerases IIα activity and epigenetic mechanisms followed by cell cycle arrest and apoptosis.

## Introduction

Hepatocellular carcinoma (HCC), the most common form of liver cancer, represent the fifth worldwide malignancy and third cause of mortality among cancer related death [1]. In Canada, the incidence of HCC has been increasing over the past several decades [2]. HCC accounts for 71.9% of liver cancers in males and females in Canada. According to Canadian Cancer Statistics in 2013, the incidence rate of liver cancer in Canada has increased by 3.6% per year, and the mortality rate increased by 2.2% per year. The contributing factors of HCC include contact with hepatocarcinogens especially aflatoxin [3], hepatic viral infection and liver cirrhosis [4]. The potential curative treatment options are surgical resection, liver transplantation, and ablation or transarterial embolization [1]. The chemotherapy, oral multikinase inhibitor sorafenib (Nexavar) is the most commonly used drug for HCC treatment but the gain in survival is modest [5]. Unavailability of effective treatments and high prevalence rate has led to the search of novel approaches suitable for prevention and treatment of liver cancer. As a result, many phytochemicals have been explored as potential chemopreventive agents that can reverse or suppress hepatocarcinogenic progression.

Flavonoids, one of the major classes of polyphenols, have shown some chemopreventive properties against HCC in a vast number of in vitro [6], [7] and in vivo studies [1], [8]. Phloridzin (phlorizin or phloretin 2′-*O*-glucoside), a dihydrochalcone, is one of the major phenolic flavonoid glucosides found in apple [9]. The major pharmacological action of phloridzin is inhibition of sodium-dependent glucose linked transporters (SGLT1 and SGLT2) that blocks intestinal glucose absorption and produce renal glycosuria [9]. In addition to their antidiabetic property, phloridzin has other biological activities such as inhibition of lipid peroxidation [10], prevention of bone loss in rats [11] and enhancement of memory in mice [12]. Phloridzin stimulate melanogenesis by increasing tyrosinase gene expression through the cAMP signaling pathway [13]. Phloretin, the aglycone of phloridzin, has been reported to have potent antioxidant activity in peroxynitrite scavenging and the inhibition of lipid peroxidation [14]. Glycosylation at 2-OH decreased the antioxidant activities of phloridzin by 18 times in comparison to phloretin [14]. Phloretin at the concentrations of 100 µM augmented TNFα-related apoptosis-inducing ligand (TRAIL)-induced apoptosis and cytotoxicity in LNCaP prostate cancer cells [15]. However, there is no report on the cancer chemopreventive effects of phloridzin. In an antitumor activity study of apple polyphenols, phloridzin did not affect the proliferation of either transplanted B16 mouse melanoma cells or BALB-MC.E12 mouse mammary tumor cells [16].

In our laboratory, phloridzin was regioselectively acylated with a series of long chain, saturated, mono- and poly-unsaturated, fatty acids by using immobilized lipase B, from *Candida antarctica* (Novozyme 435) [17]. Lipase catalyzed esterification and transesterification of flavonoid glycosides have been reported to increase lipophilicity and improved anticancer effect of the parent compound [18]. Therefore, in this study, we investigated the cytotoxic potential of fatty acid esters of phloridzin on cell proliferation of solid tumours such as hepatocellular carcinoma HepG2 cells and breast adenocarcinoma MDA-MB-231 cells as well as acute monocytes leukemia THP-1 cells. Normal human hepatocytes HP-F and rat hepatocytes RTCP10 were also used to determine the specificity of the esters on cancerous cells. This is the first time these novel fatty acid esters of phloridzin have been tested for antiproliferative effect of cancer cells. In addition to elucidate the cellular and molecular mechanisms of fatty acid esters of phloridzin on HepG2 cells, DNA topoisomerases IIα activity, cell cycle arrest, mitochondrial membrane permeability, caspase 3 activity and associated apoptotic processes were also investigated. Furthermore, we analyzed the effect of decosahexaenoic acid (DHA) ester of phloridzin on expression of 84 genes that targets for anticancer therapeutics and drug development. Our results provided experimental evidence to support further investigation of fatty acid esters of phloridzin especially DHA ester of phloridzin as an effective and safe chemotherapeutic candidate.

## Materials and Methods

### Test compounds and chemicals

Fatty acid esters of phloridzin (Pz) viz. stearic acid ester of Pz (Pz-stearic acid), oleic acid ester of Pz (Pz-oleic acid), linoleic acid ester of Pz (Pz-linoleic acid), α-linolenic acid ester of Pz (Pz-α-linolenic acid), DHA ester of Pz (Pz-DHA) and eicosapentaenoic acid ester (EPA) of Pz (Pz-EPA) were synthesised in our laboratory as previously reported [17].

Phloridzin, phloretin, caspase 3 colorimetric assay kit, propidium iodide, fatty acids namely oleic acid, stearic acid, linoleic acid, α-linolenic acid, EPA and DHA were purchased from Sigma-Aldrich Canada. Cell Titer 96 Aqueous One solution cell proliferation (MTS) assay, CytoTox 96 non-radioactive cytotoxicity (LDH) assay and CellTiter-Glo luminescent assay kits were purchased from Promega, Madison, WI, USA. Sterile dimethyl sulfoxide (DMSO) (ATCC), GFP-certified apoptosis/necrosis detection kit for microscopy from Enzo Life Sciences, Brockville, ON, Canada; ApoTarget Quick Apoptotic DNA Ladder Detection kit from Invitrogen, Burlington, ON, Canada; DCFDA-Cellular Reactive Oxygen Species detection assay kit form Abcam, Toronto, ON, Canada; and 5′,6,6′-tetrachloro-1,1′,3,3′-tetraethylbenzimidazolylcarbocyanine iodide (JC-1) from Cayman Chemicals, Burlington, ON, Canada were also used for the study.

### Cell lines and culture conditions

Human hepatocellular carcinoma cells (HepG2) and THP-1 acute monocytic leukemia cells were purchased from the American Type Culture Collection (ATCC), Manassas, VA, USA (Dalhousie University Biosafety certificate number for use of cell lines is of 2013-10). HepG2 cells were grown in Eagle’s modified minimum essential media (EMEM) supplemented with 10% FBS (ATCC) and 1% penicillin-streptomycin (ATCC). THP-1 cells were cultured in RPMI-1640 media supplemented 0.05 mM 2-mercaptoethanol and 10% fetal bovine serum to a final concentration of 10%. MDA-MB-231 breast cancer cells (ATCC HTB-26) were obtained from Cedarlane, Berlington, ON, Canada) and were maintained in DMEM medium (Sigma-Aldrich Canada) supplemented with 100 u/mL penicillin, 100 µg/mL streptomycin, 2 mM L-glutamine, 5 mM HEPES (pH 7.4) and 10% heat-inactivated fetal bovine serum (Invitrogen, Burlington, ON, Canada). Cryopreserved normal human hepatocytes (HP-F), hepatocyte plating medium and hepatocyte maintenance medium were purchased from Zen-Bio, Research Tiangle Park, NC, USA. Normal human hepatocytes plated on 96 well collagen 1 coated cell culture plates (Life Technologies) and maintained in hepatocyte maintenance medium for 24 h to allow for cell recovery and attachment. Rat hepatocytes (RTCP10), thawing media and incubation media were purchased from Life Technologies. Rat hepatocytes were plated in collagen 1 coated 96 well plates (Life Technologies, Burlington, ON, Canada) using thawing media and maintained in incubation medium. All cell types were maintained at 37°C in an incubator under 5% CO_2_/95% air atmosphere at constant humidity.

Cells were counted using a hemocytometer (Bright-Line Hemocytometer, Sigma-Aldrich Canada) and were plated according to the number of cells for each experiment in 6, 24 or 96 well format for 24 h prior to addition of test samples. All the test samples were solubilised in sterile filtered DMSO (<0.5% in the culture medium) prior to addition to the culture media. Control cells were also run in parallel and subjected to the same changes in media with <0.5% DMSO.

### Cell Proliferation assay

Cell viability was determined by using the MTS assay. In brief, HepG2 cells (5×10^3^ cells/100 µL/well), MDA-MB-231 (5×10^3^ cells/100 µL/well), THP-1 (25×10^3^/100 µL/well), normal human and rat hepatocytes (1×10^4^ cells/100 µL/well) were plated in triplicate, in a 96 well sterile flat bottom tissue culture plates. After 24 h incubation, phloridzin fatty esters, phloridzin, phloretin, free fatty acids of respective esters or sorafenib were prepared in media and 100 µL of each treatment was added to each well, each treatment in three replications. Thereby, cells were exposed to various concentrations (0.1, 1, 10, 25, 50, 75, 100 µM) of each treatment. Controls consist of cells with media containing DMSO (<0.5%), test blank wells contained the test compound in media with no cells and blank wells contained media with no cells. After additional 3, 6, 12, 18 or 24 h, 20 µL of the MTS reagent in combination with the electron coupling agent, phenazine methosulfate were added to the wells and cells were incubated in a humidified incubator for 3 h. Absorbance at 490 nm (OD490) was measured by using a Flurostar Optima microplate reader (BMG Labtech, Cary, NC, USA) to obtain the number of viable cells relative to the control population. Percentage of viability of the test compound treated cells are expressed as percentage compared to control (<0.5% DMSO). EC_50_ values (concentration required to reduce cells viability by 50% as compared to control cells) for each test compound was analysed using Graphpad Prism software, La Jolla, CA, USA. The selective index (SI) of the fatty acid esters of phloridzin is defined as the ratio of cytotoxicity (EC_50_ values) on normal HP-F cells to solid cancer HepG2, MDA-MB-231 cells (SI = EC_50_ on HP-F cells/EC_50_ on solid cancer cells). Samples with SI values greater than three were considered to have high selectivity towards cancer cells.

### Cytotoxicity assay

Lactate dehydrogenase (LDH) is a stable cytosolic enzyme that is released upon membrane damage in apoptotic/necrotic cells. LDH activity was measured using CytoTox 96 Non-Radioactive Cytotoxicity Assay (Promega, Madison, WI, USA), in which LDH released in culture supernatants is measured with a coupled enzymatic assay, resulting in conversion of a tetrazolium salt into a red formazan product. HepG2 cells (5000/100 µL/well) were seeded and treated with 100 µM of phloridzin fatty acid esters, phloridzin, phloretin, free fatty acids of respective esters or sorafenib prepared in serum free media and incubated (37°C, 5% CO_2_) for 6 h. After centrifugation, the supernatant was removed to an assay plate, and the LDH released from the cells into culture medium was measured. The maximal release was obtained after treating control cells with 1% Triton X-100 for 30 min at room temperature. The apoptotic/necrotic percentage was expressed using the formula: (sample value/maximal release)×100%. Previous studies by the supplier had clearly stated that in HepG2 cells, LDH activity maximum concentration is at 1 to 6 h incubations because LDH activity released from cells has a half-life of approximately 9 h [19]. MTS assay results showed that all the fatty acid esters of phloridzin inhibited 70–80% cell proliferation in 6 h, therefore, we analysed LDH activity after 6 h incubation.

### Morphological observation of apoptosis in HepG2 cells

#### 2.5.1. Morphologic observation under inverted phase contrast microscope

HepG2 cells were equally seeded in 24-well flat bottom tissue culture treated plates (BD Biosciences), and then treated with 100 µM of fatty acid esters of phloridzin, phloridzin, phloretin, sorafenib and DMSO (<0.5%) control. After 24 h of treatment, the morphology of HepG2 cells was observed under an inverted phase contrast microscope (Nikon Eclipse E 100, Nikon, Mississauga, ON, Canada) and were captured at 400X magnification using Infinity digital microscopy camera (Lumenera corporation, Ottawa, ON, Canada).

#### 2.5.2. Determination of Apoptosis/Necrosis by Fluorescence Microscopy

HepG2 cells were seeded into Nunc Lab-Tek two chamber slide (Sigma-Aldrich Canada) at a density of 1×10^6^ cells/chamber. The attached cells were then treated either with 100 µM fatty acid esters of phloridzin, phloridzin, phloretin, sorafenib or DMSO vehicle (as control) for 24 h. The slides were washed with diluted phosphate buffered saline. After removing the chamber, each slide was added with 50 µL of Dual Detection Reagent containing apoptosis detection reagent (Annexin V-EnzoGold) and necrosis detection reagent (7-AAD) in 1X binding buffer. The samples were incubated at room temperature for 15 min in the dark. After staining, the cells were washed with binding buffer and covered with a glass coverslip. The stained cells were observed under a fluorescence Zeiss Axiovert 200 m inverted microscope (Carl Zeiss, Toronto, ON, Canada) at magnification of ×40 with a filter set for Annexin V-EnzoGold (Ex/Em: 550/570 nm) and 7-AAD (Ex/Em: 546/647 nm).

### Analysis of apoptosis by DNA Fragmentation

HepG2 (1×10^5^) cells were seeded in 24-well culture plates and were allowed to adhere overnight. Following this, cells were treated either with 100 µM fatty acid esters of phloridzin, phloridzin, phloretin, sorafenib or DMSO vehicle (as control). The plates were re-incubated for another 24 h. DNA fragmentation was detected using ApoTarget Quick Apoptotic DNA Ladder Detection Kit (Invitrogen, Burlington, ON, Canada) according to the manufacturer’s protocol. The principle involves detecting the internucleosomal DNA fragments formed during apoptosis. Briefly, floating dead cells and trypsinized adherent cells were collected and centrifuged at 1,000 rpm for 10 min. After washing with diluted phosphate buffered saline, the cells were lysed with 35 µL TE lysis buffer (a kit component). To the lysate, 5 µL of Enzyme A (a kit component) was added and incubated at 37°C for 10 min. Afterwards, 5 µL of Enzyme B (a kit component) was added, gently mixed and incubated at 50°C for 30 min. The DNA was precipitated with the ammonium acetate and absolute ethanol at −20°C. After centrifugation (10 min at 12,000 rpm) and air drying, the DNA pellet was dissolved in 30 µL of DNA suspension buffer. For detecting the DNA ladder, the extracted DNA samples were run on a 1.2% agarose gel containing 0.5 µg/mL gel red in Tris-Borate-EDTA (TBE) buffer. After electrophoresis, the gel image was captured using Gel Doc 100 system (Bio-Rad, Mississauga, ON, Canada).

### Assay of Caspase-3 Activity

The activity of caspase 3 enzyme was measured using caspase 3 colorimetric assay kit purchased from Sigma-Aldrich. HepG2 cells (2×10^6^ cells/well), grown in 6-well plates, were treated either with 100 µM fatty acid esters of phloridzin, phloridzin, phloretin, sorafenib or DMSO vehicle (as control). Cells were lysed and the protein content of cell lysate was quantified by the BCA protein assay (Thermo Fisher Scientific Inc., Ottawa, ON, Canada). Caspase-3 activity was measured in the 200 µg of cell lysate using the caspase-specific peptide substrate, DEVD (Asp-Glu-Val-Asp), conjugated to reporter p-nitroanaline (ρ-NA) molecules. Cleavage of this peptide by caspase releases the chromophore which is measured colorimetrically at a wavelength of 405 nm as described in the supplier’s protocol.

### Cell Cycle Analysis

HepG2 cells were plated at 5×10^5^ cells per ml in a six-well plate. After 24 h incubation (37°C, 5% CO_2_), the cells were treated with 100 µM fatty acid esters of phloridzin, phloridzin, phloretin, sorafenib or DMSO (<0.5%) control prepared in media and incubated for additional 24 h. Following trypsinization, cells were washed and centrifuged at 2000×g for 10 min and the pellet re-suspended in 0.5 mL PBS. Fixation was completed by adding 1.2 mL of 70% cold ethanol for 2 h. The fixed cells were washed with PBS and centrifuged at 2000×g for 10 min. After suspending cells in 0.3 mL PBS, 8 µL of DNAase free RNAse (10 mg/mL) was added and incubated for 1 h. After adding, 15 µL of propidium iodide (0.5 mg/mL), cells were incubated in 4°C for 30 min. The cells were analyzed for cell cycle using flow cytometer FACS calibur (Beckman Coulter, Fullerton, CA, USA) with an excitation wavelength of 488 nm and emission at 670 nm. DNA content was determined by ModFit software (Verity Software House, Topsham, ME, USA), which provided histograms to evaluate cell cycle distribution.

### Mitochondrial Energy Metabolism Assays

#### ATP level assay

Cellular ATP levels were measured with CellTiter-Glo luminescent assay kit obtained from Promega according to the manufacturer’s instructions. HepG2 cells plated on a black walled clear bottom 96-well plate were incubated with 100 µM fatty acid esters of phloridzin, phloridzin, phloretin, sorafenib, free fatty acids or DMSO (<0.5%) control in media. After 24 h, CellTiter-Glo Reagent equal to the volume of cell culture medium present in each well and mixed contents for 2 min on an orbital shaker to induce cell lysis. Luminescence was recorded on Flurostar Optima microplate reader (BMG Labtech) after incubation at room temperature for 10 min to stabilize luminescent signal. The level of ATP in a sample was presented as percentage compared to untreated control.

#### Mitochondrial membrane potential (MMP)

HepG2 cells were seeded in a black walled clear bottom 96-well sterile flat bottom tissue culture plates (BD Biosciences, USA) at a density of 5×10^4^ cells/well (100 µL) and incubated in a CO_2_ incubator for 24 h at 37°C. Cells were treated with 100 µM fatty acid esters of phloridzin, phloridzin, phloretin, sorafenib, free fatty acids or DMSO (<0.5%) control prepared in media and incubated for 24 h. The staining solution JC-1 was prepared with PBS and 5 µM was added to each well. The cells were further incubated in a CO_2_ incubator at 37°C for 1 h. After washing the plate with PBS twice, the fluorescence was measured using a Fluostar Optima microplate reader (BMG Labtech) at 535 nm for JC-1 monomers and at 590 nm for JC-1 aggregates.

### Human Topoisomerase IIα (topo IIα) Catalytic Activity

The topo IIα catalytic activity was monitored via electrophoresis using topoisomerase II drug screening kit (TopoGEN, Inc., Columbus, OH, USA). Briefly, 20 µL of reaction mixtures contained 0.5 M Tris-HCl, pH 8.0, 1.5 M NaCl, 100 mM MgCl_2_, 20 mM ATP, 300 µg BSA/mL and 5 mM dithiothreitol. Supercoiled DNA (pHOT1 DNA), supercoiled provided in the kit was determined to be ideal for this assay because it is small and easy to handle and has a large number of topo IIα recognition elements. After 2 µL (0.25 µg) of pHOT1 DNA was added, followed by the addition of 100 µM fatty acid esters of phloridzin, phloridzin, phloretin, sorafenib or DMSO (<0.5%) control in solvent, the reaction was initiated by adding 4 units (2 µL) of human DNA topo IIα and carried out at 37°C for 30 min. The reaction was terminated by adding 2 µL of 10% sodium dodecyl sulfate (SDS) followed by digestion with 2 µL of proteinase K (50 µg/mL) at 37°C for 15 min to degrade enzyme. After adding 2 µL of loading buffer (0.25% bromophenol blue and 50% glycerol) was added to the mixture, samples were loaded onto 1% agarose gel. Electrophoresis was conducted at 66 V (2 V/cm) for 5 h in TBE buffer using Biorad Electrophoretic Gel System (Bio-Rad, Hercules, CA, USA). Supercoiled DNA (pHOT1 DNA) and relaxed DNA were included in the electrophoresis run as markers for DNA topology. Gels were then stained in 0.5 µg/mL gel red in TBE for 30 min and destained for 15 min in distilled water prior to digital image acquisition using Gel Doc 100 system (Bio-Rad, Hercules, CA, USA).

One unit of topoisomerase II activity was defined as the minimum amount of enzyme required to achieve complete relaxation of 0.5 mg superhelical pHOT1 DNA in 30 min at 37°C. Inhibition of topoisomerase II relaxation activity was investigated by the same procedure using four units of enzyme and 100 µM test compounds. The percent of inhibition was calculated by the following formula:
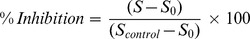
Where S_control_ is the percent of supercoiled DNA in the control lane (without enzyme and test compounds), S_0_ is the percent of supercoiled DNA in the lane without test compounds and S is the percent of supercoiled DNA in the lane with test compounds and enzyme.

### Real Time RT-PCR analysis

Gene expression profiles were obtained from HepG2 cells treated with DHA esters of phloridzin or sorafenib or DMSO treated control cells. Total RNA extraction was performed using Arum Total RNA minikit (Bio-Rad, Hercules, CA, USA). RNA concentration and purity was determined by measuring the absorbance using a NanoDrop (NanoDrop Technologies, Wilmington, DE, USA). RNA integrity was assessed by formaldehyde agarose gel electrophoresis. RNA (400 ng) was used to synthesize cDNA using RT^2^ First Strand kit (SABiosciences, Frederick, MD, USA). RT^2^ RNA QC PCR arrays (SABiosciences, Frederick, MD, USA) was used to assess the quality of cDNA samples before characterization with the human cancer drug targets RT^2^ profiler PCR array (SABiosciences, Frederick, MD, USA). Gene expression profiles of 84 genes were investigated using the human cancer drug targets RT^2^ profiler PCR array (PAHS-507ZD) on a Bio-Rad CFX Connect (Bio-Rad, Hercules, CA, USA) using RT^2^ real-time SYBR green PCR master mix (SABiosciences, Frederick, MD, USA). The array also has five reference genes (beta-2-microglobulin (B2M), hypoxanthine phosphoribosyltransferase 1 (HPRT1), ribosomal protein L13a (RPL13A), glyceraldehyde-3-phosphate dehydrogenase (GAPDH), and actin beta (ACTB), three reverse transcription controls (RTCs), three positive PCR controls (PPCs), and one genomic DNA control (GDC), making up to 96 assays. After normalization with RPL13A reference gene, gene expression levels were individually assessed by using the threshold cycle (Ct) values using RT^2^ profiler PCR array data analysis software (Microsoft Excel-based program of SABiosciences, Mississauga, ON, Canada). It calculates: 1) ΔCt of each gene = Ct of gene of interest - average Ct of chosen reference genes 2) ΔΔCt for each gene across two groups; ΔΔCt = ΔCt (Pz-DHA ester or sorafenib) - ΔCt (control) & 3) fold-change for each gene from control group to Pz-DHA ester treated group as 2 ^(−ΔΔCt)^. RT^2^ RNA QC PCR data showed no genomic DNA contamination (Ct <35 will indicate least GDC) or presence of impurities in RNA samples based on the Ct value of PPC (Ct should be 20±2 on each array) and showed no inhibition of reverse transcription based on the Ct values of RTC and PPC. Reproducibility was maintained by using three biological replicates from three individual experiments.

### Statistical analyses

EC_50_ values were calculated using Graphpad Prism 6 software (GraphPad Software Inc., San Diego CA, USA). Statistical analysis was performed using Statistical Analysis System (SAS, Version 9.2). One-way ANOVA with Tukey’s post hoc comparisons at P<0.001 was used for statistical comparisons. All data are presented as a mean value with its standard deviation indicated (Mean ± SD).

## Results

### Antiproliferative effect of fatty acid esters of phloridzin in various cancer and normal cells

**Figure 1:**
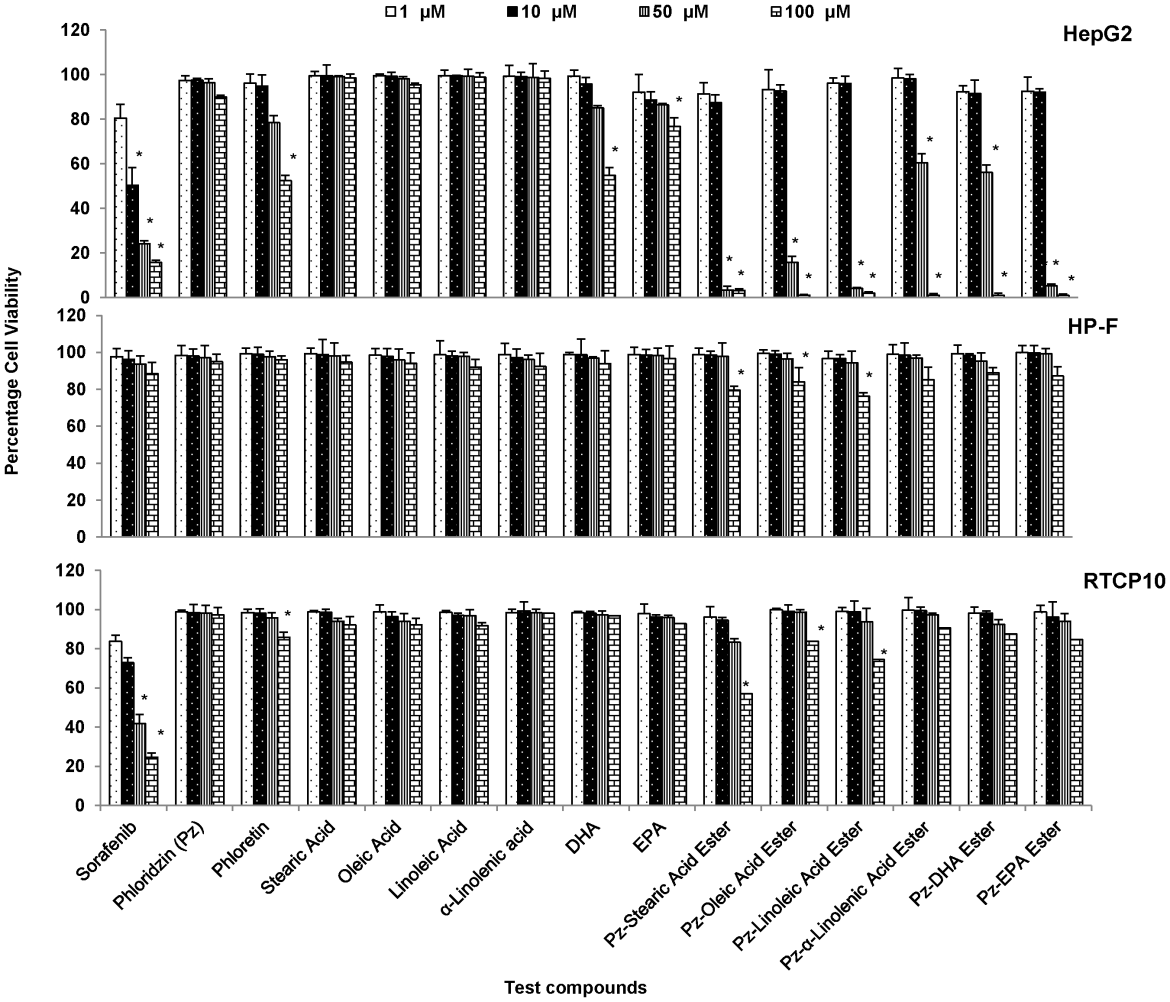
Antiproliferative effect of fatty acid esters of phloridzin on HepG2 and normal cells. Hepatic carcinoma (HepG2) cells and normal human hepatocytes (HP-F) and rat hepatocytes (RTCP10) cells were exposed to test compounds at 1, 10, 50, 100 µM for 24 h. The cell viability was determined using MTS assay. The data presented as the percentage viability relative to vehicle only treated control group. Data are presented as the mean ± SD (n = 3) are representative of at least three separate independent experiments. *P<0.05 significantly different from the vehicle only control group (Tukey HSD, P<0.01).

In the present study, the potential cytotoxic effects of fatty acid esters of phloridzin, phloridzin, free fatty acids and phloretin as well as standard commercial cancer drugs were investigated on human hepatocarcinoma (HepG2), breast adenocarcinoma (MDA-MB-231) and acute monocytic leukemia (THP-1) cell lines, primary normal human hepatocytes and rat hepatocytes by using MTS assay. The assay showed that fatty acid esters of phloridzin kills HepG2, MDA-MB-231 and THP-1 cells to a similar extent and in a dose- (Figure 1) and time-dependent manner (Table 1). After 24 h incubation, in HepG2 cells, antiproliferation EC_50_ for stearic acid, oleic acid, linoleic acid, α-linolenic acid, DHA and EPA esters of phloridzin were 37.8, 31.5, 29.2, 53.1, 51.9 and 26.8 µM respectively. EC_50_ were 35.2, 37.9, 32.3, 63.8, 55.5, and 26.5 µM in MDA-MB-231 cells. EC_50_ values of these esters on THP-1 cells were 40.7, 2.1, 6.2, 35.7, 27.3, and 14.8 µM. Although fatty acid esters of phloridzin showed high potency as antiproliferative agent, none of the parent molecule, phloridzin and individual fatty acids showed any effect on cell viability (EC_50_>100 µM) of HepG2, MDA-MB-231 or THP-1 cells. Interestingly, aglycone phloretin showed a significant antiproliferative effect (EC_50_ 39.6 µM) in THP-1 cells (Table 1).

**Table 1:** EC_50_± SD values of tested compounds in three human cancer cell lines and a normal cells (HP-F).

		EC_50_ (µM)															
		HepG2					MDA-MB-231					THP-1					HP-F
		3 h	6 h	12 h	18 h	24 h	3 h	6 h	12 h	18 h	24 h	3 h	6 h	12 h	18 h	24 h	24 h
**Phloridzin** **(Pz) Esters**	**Pz-Oleic acid ester**	64.6±5.1	60.9±4.9	54.2±4.8	48.5±4.0	37.8±3.1(4.7)^Z^	63.4±5.8	57.4±3.9	50.5±4.1	37.4±2.9	35.2±2.3(5.1)	95.8±8.1	61.8±5.1	53.8±4.9	41.7±3.8	40.7±2.9(4.4)	>100
	**Pz-Stearic acid** **ester**	53.3±4.5	48.2±3.9	37.6±1.8	34.8±2.0	31.5±2.1(4.2)	64.8±5.4	61.1±5.5	57.4±4.6	55.7±4.8	37.9±2.1(3.5)	39.2±3.4	32.3±1.8	23.9±1.7	22.9±1.8	2.1±0.5(64)	>100
	**Pz-Linoleic acid** **ester**	43.3±2.9	40.5±3.1	35.4±2.2	30.8±2.7	29.2±1.9(4.5)	41.6±2.9	38.8±2.0	38.1±2.5	36.1±2.6	32.3±1.9(4.1)	36.6±2.1	18.7±1.1	14.7±0.9	13.1±0.9	6.2±0.4(21.2)	>100
	**Pz-α-Linolenic** **acid ester**	76.5±6.0	66.3±5.2	60.5±4.8	55.7±4.1	53.1±3.9(3.14)	89.9±6.8	83.8±6.7	80.5±7.1	66.1±5.4	63.8±5.1(3.1)	88.7±6.7	72.4±6.0	41.3±2.8	37.9±2.1	35.7±1.7(4.67)	>100
	**Pz-DHA ester**	76.9±5.8	64.4±5.9	56.2±4.9	54.2±3.9	51.9±3.8(11.2)	131.5±9.9	83.7±7.5	70.2±6.1	62.6±5.1	55.5±4.5(10.5)	65.8±5.2	49.5±3.8	42.7±3.0	35.8±2.8	27.3±1.2(21.3)	>100
	**Pz-EPA ester**	39.4±2.8	37.8±1.9	36.0±2.1	33.1±2.4	26.8±1.8(7.4)	37.9±2.2	33.6±1.8	32.6±1.8	31.5±2.3	26.5±1.1(7.5)	27.9±1.5	19.9±1.1	17.0±0.6	15.8±0.8	14.8±0.6(21.38)	>100
**Parent** **Compounds**	**Phloridzin**	>100	>100	>100	>100	>100	>100	>100	>100	>100	>100	>100	>100	>100	>100	>100	>100
	**Oleic acid**	>100	>100	>100	>100	>100	>100	>100	>100	>100	>100	>100	>100	>100	>100	>100	>100
	**Stearic acid**	>100	>100	>100	>100	>100	>100	>100	>100	>100	>100	>100	>100	>100	>100	>100	>100
	**Linoleic acid**	>100	>100	>100	>100	>100	>100	>100	>100	>100	>100	>100	>100	>100	>100	>100	>100
	**α-Linolenic acid**	>100	>100	>100	>100	>100	>100	>100	>100	>100	>100	>100	>100	>100	>100	>100	>100
	**DHA**	>100	>100	>100	>100	>100	>100	>100	>100	>100	>100	>100	>100	>100	>100	>100	>100
	**EPA**	>100	>100	>100	>100	>100	>100	>100	>100	>100	>100	>100	>100	>100	>100	>100	>100
**Drugs**	**Sorafenib**	67.5±5.2	46.7±3.5	17.3±0.8	13.4±0.9	10.7±0.5											>100
	**Doxorubicin**						>100	>100	9.6±0.2	5.9±0.3	4.1±0.1						
	**Daunorubicin**											48.1±2.8	35.2±2.9	12.6±0.8	1.4±0.2	0.02±0.01	
**Aglycone of** **Phloridzin**	**Phloretin**	>100	>100	>100	>100	97.20	>100	>100	>100	>100	>100	73.5±5.2	64.1±5.4	60.1±4.2	56.9±3.0	39.6±1.7	

Z Numbers in the parenthesis are SI values, whereas SI = EC_50_ of HP-F cells/EC_50_ on solid cancer cells.

To evaluate the specificity of fatty acid esters of phloridzin to cancer cells, drug effect on cell viability in normal hepatocytes was quantified by cytotoxicity assay in both normal human (HP-F) and rat (RTCP10) hepatocytes. HP-F cells were treated with 100 µM and lower concentrations of all fatty acid esters of phloridzin, phloridzin, fatty acid, sorafenib and phloretin for 24 h (Table 1). Fatty acid esters of phloridzin did not affect the viability of normal human hepatocytes with EC_50_>100 µM and are more specific to cancer cell lines (Table 1, Figure 1). In the 100 µM treatment of fatty acid esters of phloridzin for 24 h, fatty acid esters of phloridzin showed least toxicity (>90% viability) in rat hepatocytes also (Figure 1). The most promising and most selective cytotoxic activities were detected in Pz-DHA and Pz-EPA esters. Fatty acid esters of phloridzin except Pz-stearic acid (about 50% viability) also showed much less activity in inhibiting cell viability (>80% viability) of rat hepatocytes than that of cancer cell lines. These results suggest that fatty acid esters of phloridzin may have moderate to minimal side effects. The most promising and most selective cytotoxic activities were detected with Pz-DHA ester. The EC_50_ (µM) and SI values of Pz-DHA in HepG2, MDA-MB-231, THP-1 were 51.9 (SI = 11.2), 55.5 (SI = 10.5), and 27.3 (SI = 21.38), respectively (Table 1). DHA is a common dietary omega-3 fatty acid and it also possesses antiproliferative properties [20]. Therefore, Pz-DHA ester was selected for gene expression study using human drug target RT^2^-PCR array as it showed the strongest cytotoxic effect on cancer cells and was the least toxic on normal cells compared to other fatty acid esters of phloridzin.

HepG2 cells were chosen as the representative cell line for the remainder of this investigation because these liver cancer cells were susceptible to killing by fatty acid esters of phloridzin and it has functionally active p53 protein, competent DNA-repair system, active enzymes for phase-I and -II metabolism which may give a high predictivity for in vivo genotoxicity.

### Increase of LDH Leakage by fatty acid esters of phloridzin

**Figure 2:**
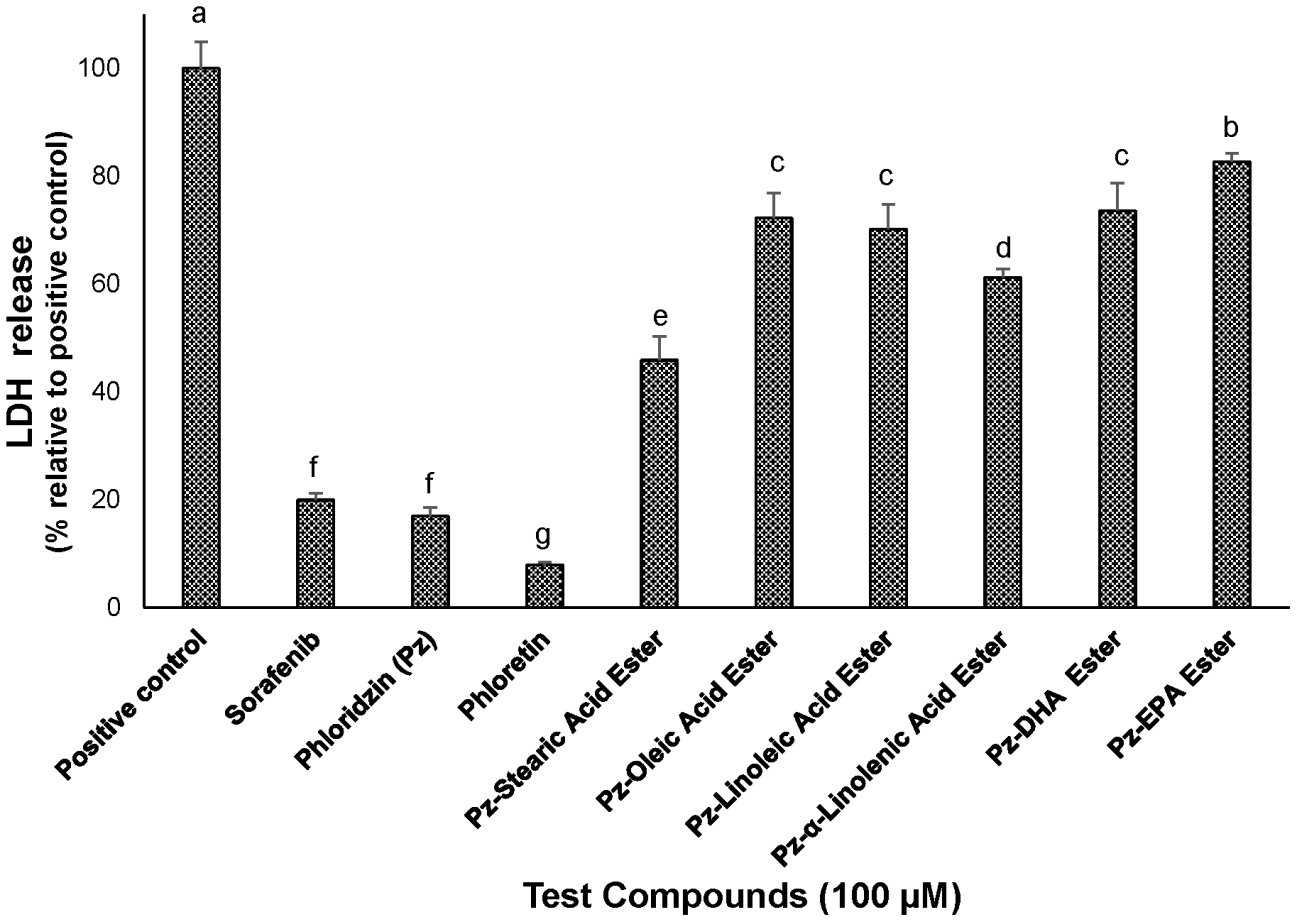
Percentage LDH release relative to control in HepG2 cells. The cells were incubated with 100 µM of fatty acid esters of phloridzin (Pz) in comparison with parent compounds phloridzin, phloretin (aglycone), or liver cancer drug sorafenib for 24 h. Data are presented as the mean ± SD (n = 3) are representative of at least three separate independent experiments. Different letters represent significantly different mean values from other treatments (Tukey HSD, P<0.01).

Fatty acid esters of phloridzin were evaluated for cytotoxic effects in HepG2 cells (100 µM) using LDH release assay. Incubating the cells with 100 µM of all six fatty esters of phloridzin resulted in significant increase in LDH activity (P<0.001) at 6 h compared to sorafenib, phloridzin and phloretin. Maximum LDH release was showed by pz-EPA ester (82.7±1.5%) and least by phloretin (7.9±0.5%) compared to the positive control (maximum release by 1% Triton-10X treated cells and incubated for 30 min). These results are agreeable with the previous results of MTS cell viability assay (Figure 2).

### Morphological evaluation of apoptosis

#### Apoptotic morphological changes of cells observed under inverted phase contrast microscope

**Figure 3:**
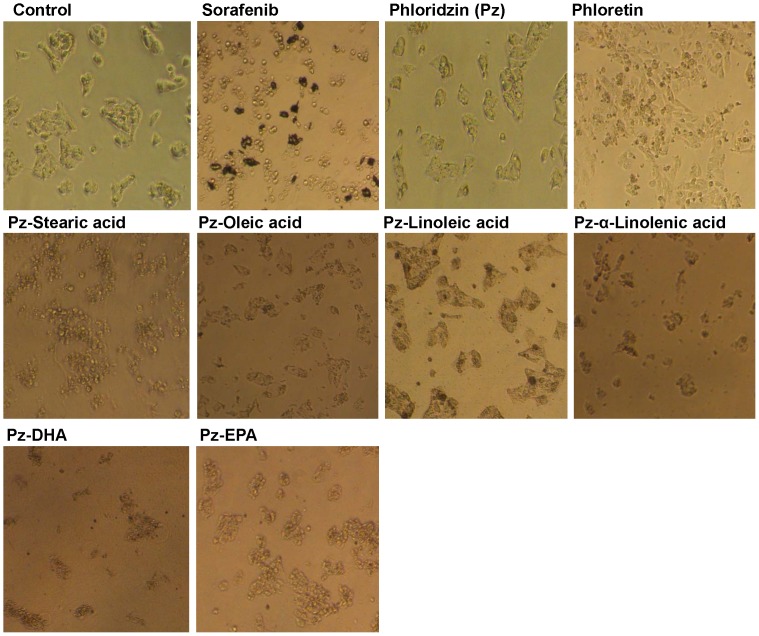
Inverted phase contrast microscopy images of HepG2 cells. The cells were cultured (2×10^4^ cells/100 µL) in 96 well plate after incubation with 100 µM of fatty acid esters of phloridzin (Pz) in comparison with parent compounds phloridzin, phloretin (aglycone) or liver cancer drug sorafenib for 24 h. Shown are representative images of three independent experiments.

Treatments with fatty acid esters of phloridzin and sorafenib (100 µM for 24 h) showed the distorted membrane structure, shrinkage of the cells and the nucleus as well as condensation of nuclear chromatin into sharply delineated masses. The cell detaches from the plate and its outlines become convoluted and form extensions. These cells showed morphological changes which are characteristic to apoptosis. Phloridzin treated cells have shown no effect and morphology was similar to the control cells (Figure 3).

### Apoptosis assessment using Annexin V/7-AAD staining assay

**Figure 4:**
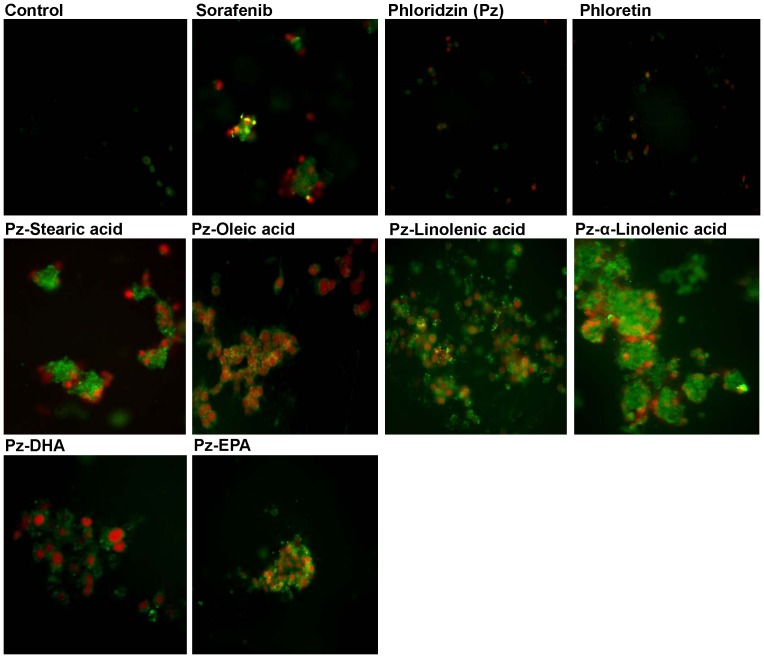
Fluorescent microscopic analysis of apoptotic HepG-2 cells stained with Annexin V-FITC and 7-AAD. The cells were cultures (1×10^5^ cells) in chambered slides and incubated with 100 µM of fatty acid esters of phloridzin (Pz) in comparison with parent compounds phloridzin or phloretin (aglycone) or liver cancer drug sorafenib. Green and red color indicates early and late apoptotic cells, respectively. Shown are representative images of three independent experiments.

Fluorescence imaging was conducted to visually differentiate between apoptosis induced and necrotic cell death. After incubation of HepG2 cells with 100 µM of fatty acid esters of phloridzin for 24 h, the number of cells remaining as an adherent monolayer was greatly decreased compared to the control cells. In addition, floating cells showed morphological changes, with characteristics similar to apoptosis or necrosis. We co-incubated cells with Annexin V Enzogold (enhanced cyanine), an early marker of phosphatidylserine externalization at the cell membrane and red emitting dye 7-AAD, marker of late apoptosis or necrosis. Upon exposure to fatty acid esters of phloridzin most of the HepG2 cells treated with fluorescent dyes stained green for Annexin V denoting apoptosis and less number of cells stained red for 7-AAD indicating late apoptosis or necrosis. DMSO (<0.5%) control, phloridzin and phloretin treated cells showed less colour indicate less apoptotic death of cells. The results suggested that fatty acid esters of phloridzin induced marked apoptotic and less necrotic morphology in HepG2 cells (Figure 4).

### Induction of internucleosomal DNA fragmentation

**Figure 5:**
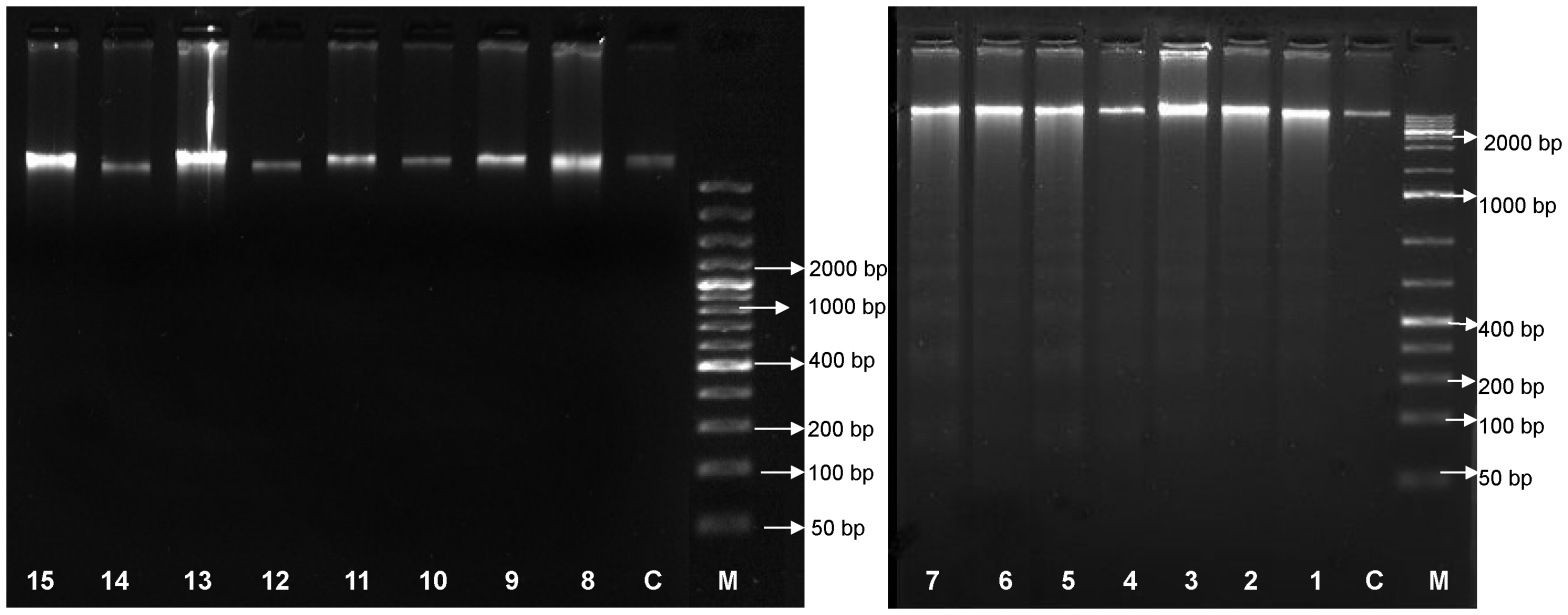
DNA fragmentation of HepG2 cells. DNA were isolated after incubation of HepG2 cells with 100 µM of fatty acid esters of phloridzin (Pz) in comparison with parent compounds phloridzin, free fatty acids, phloretin (aglycone) or liver cancer drug sorafenib for 24 h. Lane M: molecular-weight marker, lane C: control, lane 1–6: stearic acid ester of Pz, oleic acid ester of Pz, linoleic acid ester of Pz, α-linolenic acid ester of Pz, DHA ester of Pz and EPA ester of Pz, lane 7: sorafenib, lane 8–13: stearic acid, oleic acid, linoleic acid, α-linolenic acid, DHA and EPA, lane 14: phloridzin, and lane 15: phloretin. Shown are representative gel images of three independent experiments.

Internucleosomal DNA fragmentation is a biochemical hallmark of apoptotic cell death. From the agarose gel, DNA samples from the fatty acid esters of phloridzin or sorafenib (100 µM) treated HepG2 cells exhibited intact genomic DNA and clear DNA ladders (Figure 5: Lane 1–7). Treatments with DMSO (<0.5%) control (C) or free fatty acids of respective esters (Lane 8 to 13) phloridzin (Lane 14) or phloretin (Lane 15) showed only intact genomic DNA and do not marked any DNA laddering or even smearing effect. The pattern of laddering of DNA from fatty acid esters of phloridzin or sorafenib treated cells are characteristic that commonly associated with apoptotic process, in which the DNA is cleaved into fragments of 180–200 base pairs (bp) by the endogenous endonucleases [21] (Figure 5).

### Caspases 3 activation

**Figure 6:**
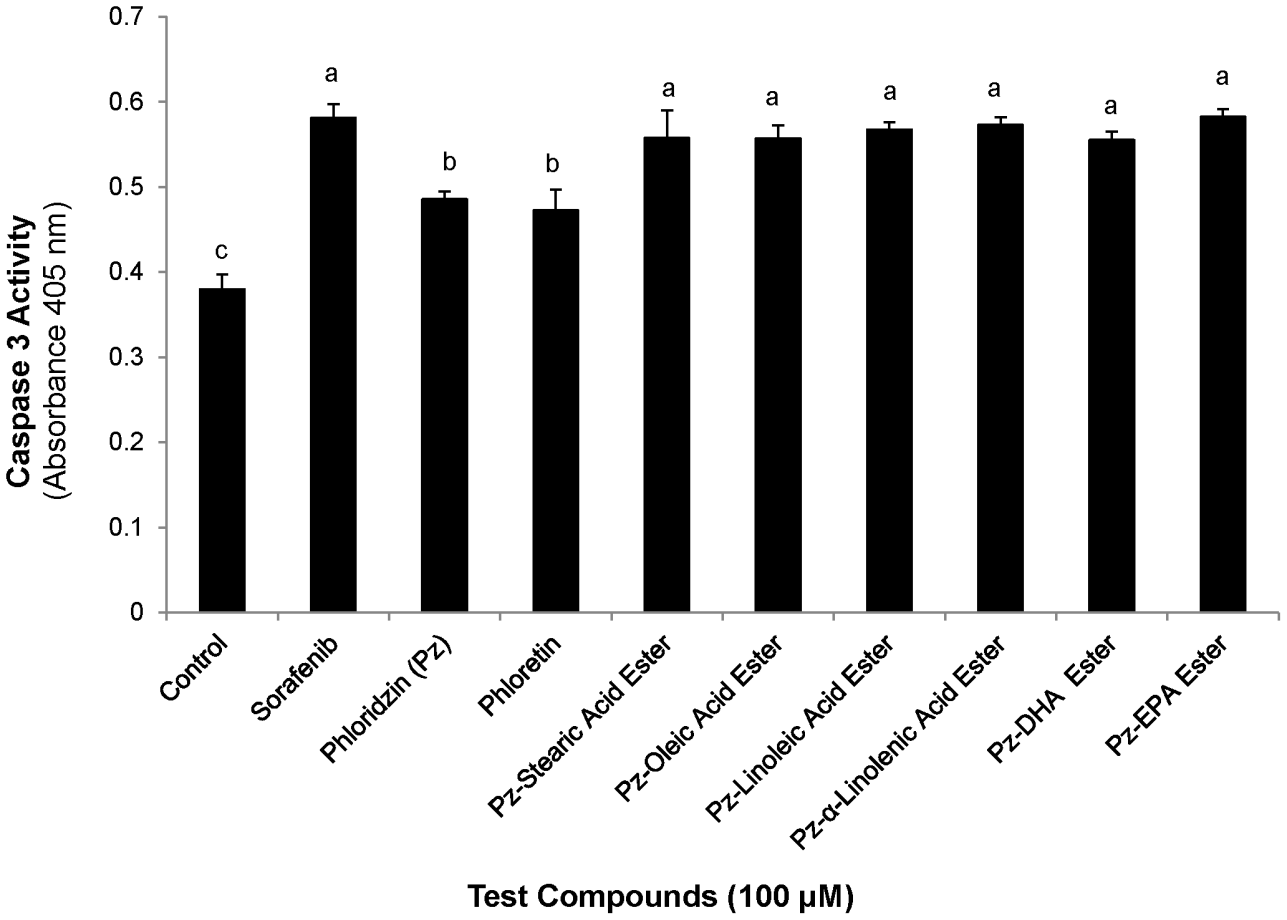
Caspase 3 activity of HepG2 cells. The cells were incubated with 100 µM of fatty acid esters of phloridzin (Pz) in comparison with parent compounds phloridzin, phloretin (aglycone) or standard drug sorafenib for 24 h. Data are presented as the mean ± SD (n = 3) are representative of at least three separate independent experiments. Different letters represent significantly different mean values from other treatments (Tukey HSD, P<0.01).

Programmed cell death is associated with induction of caspases which finally lead to the disruption of cellular structures. Incubation of HepG2 cells with fatty esters of phloridzin or sorafenib for 24 h induced a significant (P<0.001) increase in caspase 3 activity compared to DMSO treated control at 24 h. These findings emonstrated that the esters induces caspase 3 activation that is required for execution of apoptotic cell death. Phloridzin and phloretin showed enhanced caspase 3 activity but significantly (P<0.001) lower than fatty acid esters of phloridzin or the drug (Figure 6).

### Fatty acid esters of phloridzin decrease mitochondrial membrane potential and ATP level

**Figure 7:**
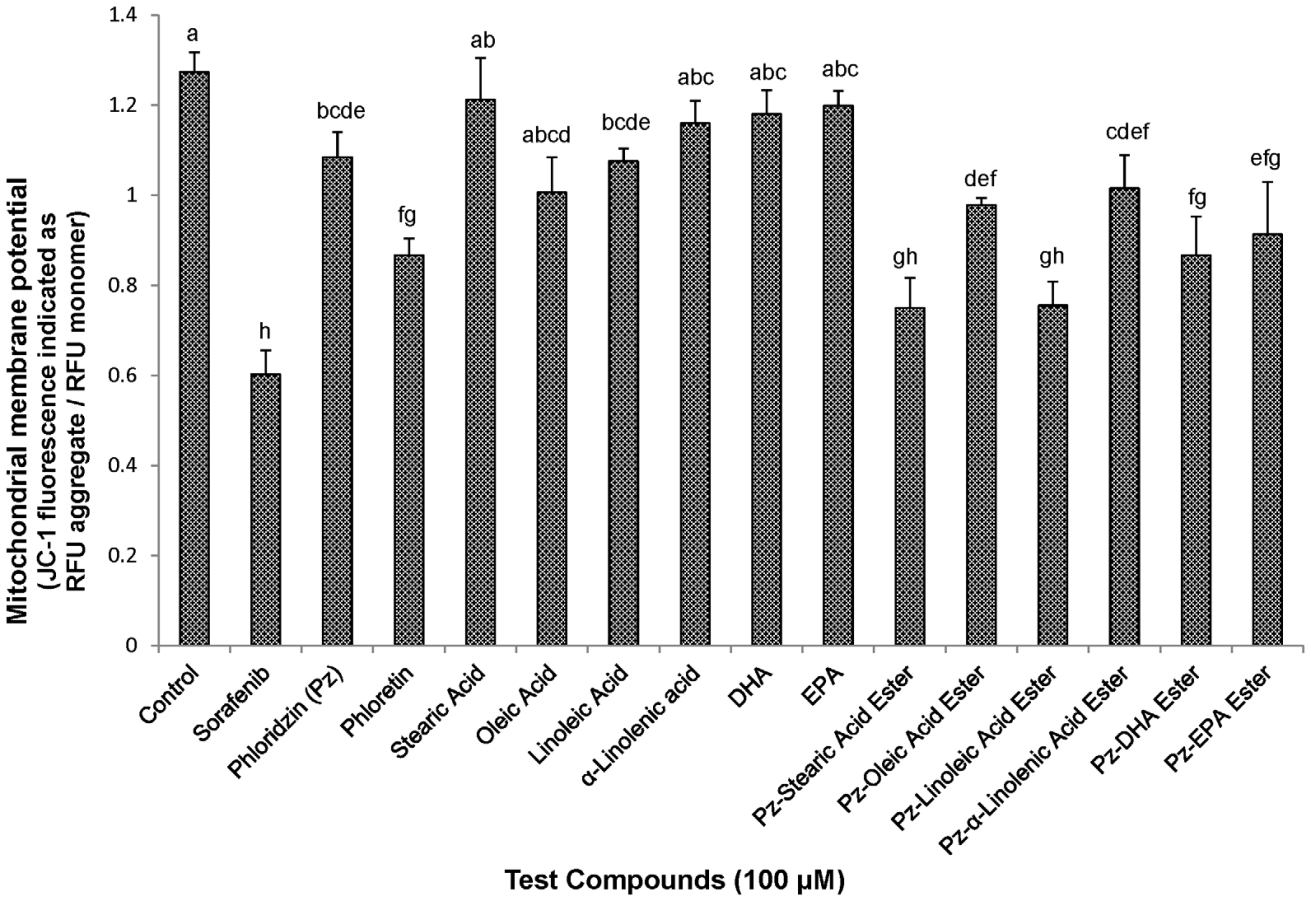
Mitochondrial membrane potential (Δψm) of HepG2 cells measured by JC-1 fluorescence. HepG2 cells were treated with 100 µM of fatty acid esters of phloridzin (Pz) in comparison with parent compounds phloridzin, individual fatty acids, phloretin (aglycone) or liver cancer drug sorafenib for 24 h. The fluorescence of JC-1 monomers was measured at em 535 nm and aggregates at em 590 nm. Data are presented as the mean ± SD (n = 3) are representative of at least three separate independent experiments. Different letters represent significantly different mean values from other treatments (Tukey HSD, P<0.01).

**Figure 8:**
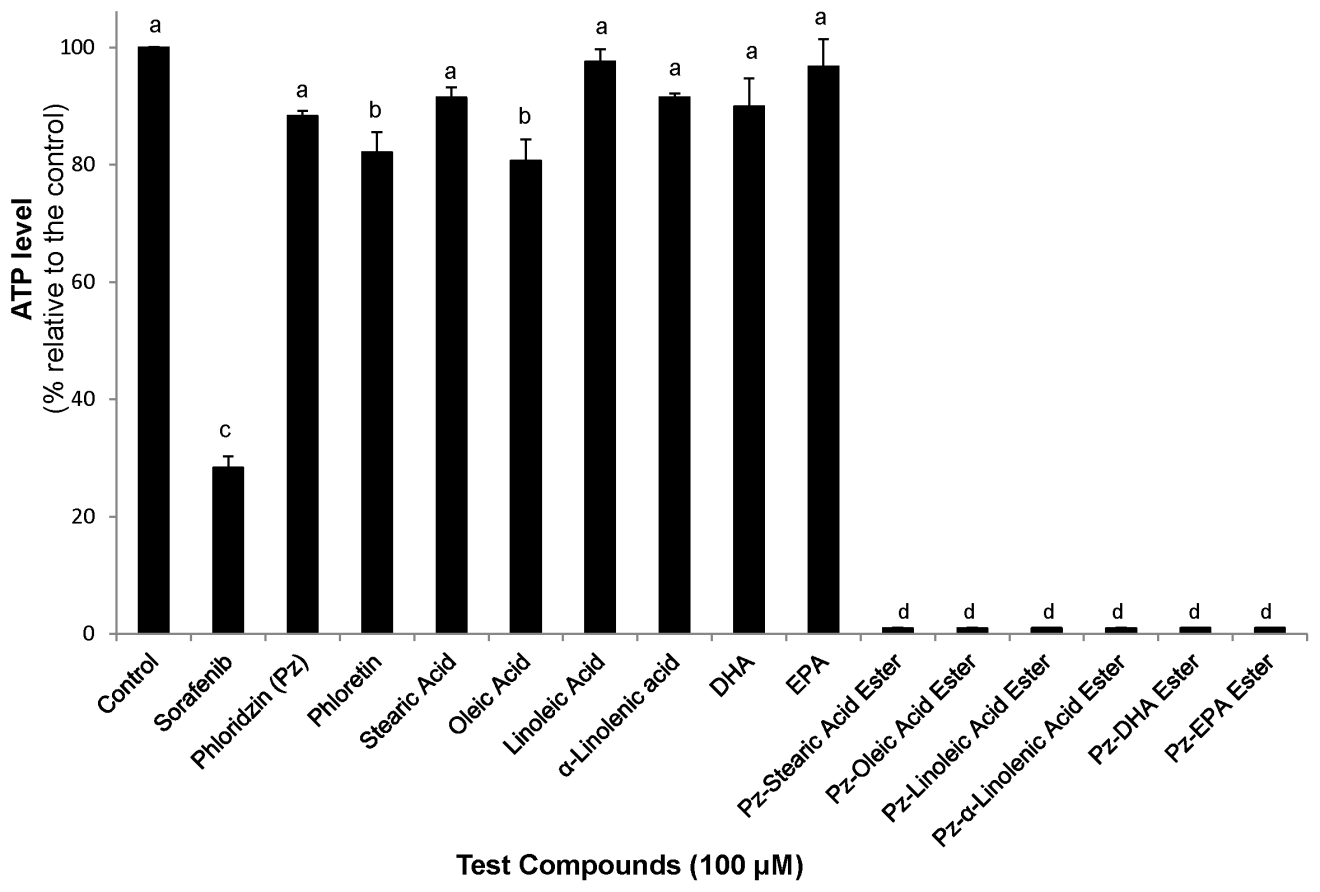
Cellular ATP level in HepG2 cells. The cells were treated with 100 µM of fatty acid esters of phloridzin (Pz) in comparison with parent compounds phloridzin, free fatty acids, phloretin (aglycone) or liver cancer drug sorafenib for 24 h. Cellular ATP content in treated cells is expressed as percentage compared to ATP levels in untreated controls. Data are presented as the mean ± SD (n = 3) are representative of at least three separate independent experiments. Different letters represent significantly different mean values from other treatments (Tukey HSD, P<0.01).

We examined whether mitochondrial events were associated with apoptosis induction by fatty acid esters of phloridzin treated cancer cells. Mitochondrial membrane potential, a reliable indicator of mitochondrial dependent apoptosis, is quantified by applying the fluorescent dye JC-1. In the presence of low mitochondrial membrane potential, the dye forms JC-1 monomers. We observed significant (P<0.05) reduction in mitochondrial membrane potential in HepG2 cells treated with fatty acid esters of phloridzin, sorafenib and phloretin compared to control and parent compounds (Figure 7). Treatment of HepG2 cells for 24 h with 100 µM fatty acid esters of phloridzin was associated with an >95% decrease in ATP levels (Figure 8). Therefore, we speculate that cellular apoptosis induced by fatty acid esters of phloridzin is a mitochondrial dependent mechanism.

### Altered cell cycle phase distributions

**Figure 9:**
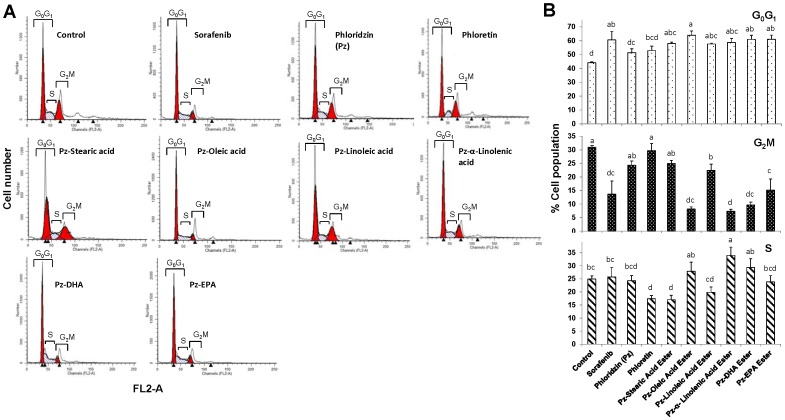
Evaluation of cell cycle in HepG2 cells using the propidium iodide assay. HepG2 cells were treated with 100 µM of fatty acid esters of phloridzin (Pz), pz or phloretin or sorafneib for 24 h, and then cell cycle profile was determined using flow cytometry after staining with PI/RNase. Representative FACS histograms of nuclear DNA content are shown (A). The DNA content peaks indicating cells in the G_0_G_1_, S, or G_2_/M phase of the cell cycle are marked. The bar chart show the changes in percentages (mean ± SD) of cells in G_0_/G_1_, S-phase, and G_2_/M after treatment (B). Data are presented as the mean ± SD (n = 3) are representative of three separate independent experiments. Different letters represent significantly different mean values from other treatments (Tukey HSD, P<0.01).

As summarized in Figure 9, treatment of HepG2 cells with 100 µM of fatty acid esters of phloridzin resulted in a significantly higher number of cells in the G_0_/G_1_ phase (52% to 60%) compared with control (48%). Potency of Pz-oleic acid, Pz-α-linolenic acid, Pz-DHA and Pz-EPA esters to induce G_0_/G_1_ phase arrest was similar to prescribed commercial drug sorafenib. As, in each case, there was a concomitant reduction in the number of cells in the S and G_2_/M phases. This experiment suggests that fatty acid esters of phloridzin induce G_0_/G_1_ phase cell cycle arrest in HepG2 cells (Figure 9).

### Catalytic inhibition of human DNA Topoisomerase II α

**Figure 10:**
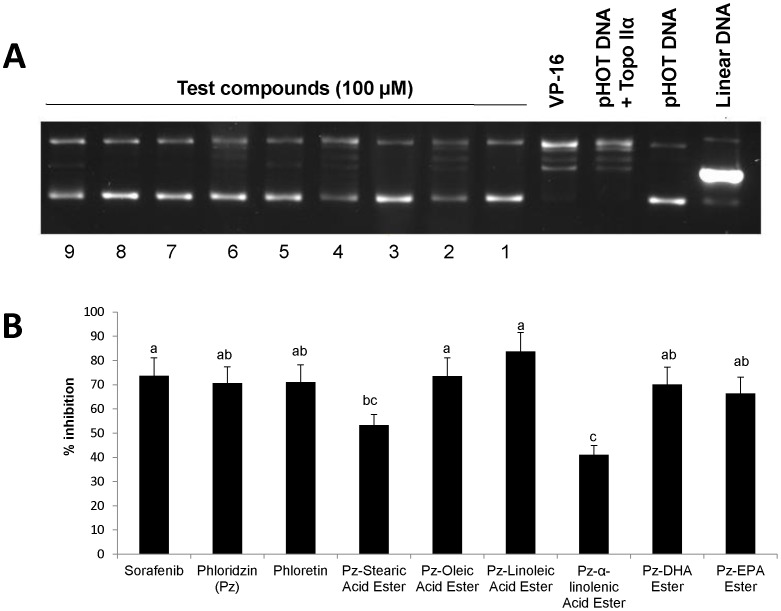
Activity of human topoisomerase II of HepG2 cells. The cells were incubated with 100 µM of fatty acid esters of phloridzin (Pz) in comparison with parent compounds phloridzin, phloretin (aglycone) or sorafenib for 24 h. A. Lanes 1 Pz-oleic acid, lane 2: Pz-stearic acid, lane 3: Pz-linoleic acid, lane 4: Pz-α-linolenic acid, lane 5: Pz-DHA, lane 6: Pz-EPA, lane 7: phloridzin, lane 8: sorafenib, and lane 9: phloretin. The data are representative of three separate, independent experiments. B. Percentage inhibition.

Fatty acid esters of phloridzin, phloridzin, sorafenib as well as phloretin inhibited catalytic activity of human DNA topo IIα. Effect of new compounds on the catalytic activity of DNA topo IIα was evaluated by supercoiled pHOT DNA. As seen in Figure 10, all the test compounds at 100 µM inhibited pHOT DNA relaxation and most of the pHOT DNA remained in supercoiled state. VP-16, an interfacial poison showed linear band and less potent than catalytic inhibition induced by phloridzin fatty acid esters. Inhibition of topo IIα is a good target for anticancer drugs (Figure 10).

### Molecular mechanisms of antiproliferative effect of Pz-DHA ester

Molecular pathogenesis of HCC is complex that involves genetic and epigenetic alterations and altered molecular pathways. Based on the least toxicity on normal hepatocytes and high selectivity on cancerous cells, Pz-DHA ester was used for further investigation involving gene expression related to cell growth. According to gene expression studies in HCC, genes responsible for cell proliferation and survival are activation of EGFR signalling, IGF signalling, Ras/MAPK signalling or AKT/mTOR signalling [22]. Evasion of apoptosis due to deregulation of intrinsic or extrinsic pathway is one of the hallmark of liver cancer [22]. To reveal possible molecular mechanisms of the antiproliferative effect of Pz-DHA ester to HepG2 cells, human cancer drug targets RT^2^ profiler PCR array was used. This system profiles the expression of 84 actively sought targets for anticancer therapeutics and drug development (http://www.sabiosciences.com/rt_pcr_product/HTML/PAHS-507Z.html). The relative expression of each gene in any two samples (experimental and control) are summarized in the Table 2 revealing genes up- and down-regulated in the experimental sample. In this study, comparing the genes with more than two-fold expression change between Pz-DHA ester treated HepG2 cells with the vehicle treated control, it has shown that the expression of 41 genes was down-regulated and 8 genes up-regulated while the expression of 35 genes were unaffected. Pz-DHA ester treatment down-regulated the expression of some cancer related genes which are represented as folds in brackets: As shown in Table 2, Pz-DHA ester treatment significantly down regulated (P<0.05) the expression of most of cancer promoting genes which are represented as folds in brackets:

**Table 2:** Genes with altered expressions in HepG2 cells treated with DHA ester of phloridzin or sorafenib.

Mechanism	Gene	Fold change of gene expression relative to control Z	
		Phloridzin-DHA	Sorafneib
Apoptosis	BCL2	−2.8	
PI-3 Kinases & Phosphatases	mTOR	−3.6	−2.1
	PIK3C2A	−2.2	
Growth Factors & Receptors	EGFR	−8.9	−2.0
	ERBB2	−5.7	
	ERBB3	−6.1	
	FLT1	+16.5	+5.5
	IGF1R	−4.2	−2.1
	IGF2	−2.3	−2.8
	KDR	−2.8	+2.0
	PDGFRA	−2.8	
	PDGFRB	−16.8	−2.5
Receptor Tyrosine Kinase Signaling	AKT1	−6.1	
	AKT2	−2.8	
	GRB2	−8.6	+2.0
Cell Cycle	CDK2	−2.0	
	CDK9	−2.1	
	MDM4	−2.1	−2.3
	TERT	−5.6	
Topoisomerases, Type II	TOP2A	−2.7	
	TOP2B	−2.3	−2.2
Transcription Factors	IRF5	−2.3	
	NF−κB	−4.3	−2.5
	TP53.	−7.4	
Protein Kinases	AURKB	−2.0	
	PLK1	−2.9	
	PRKCA	−8.1	
	PRKCD	−2.1	
	PRKCE	−5.6	
RAS Signaling	KRAS	−4.0	
	NRAS		−2.4
Histone Deacetylases	HDAC1	−2.6	
	HDAC11	−20.1	
	HDAC4	−4.1	
	HDAC6	−5.4	
	HDAC7	−6.0	
Poly ADP-Ribose Polymerases	PARP1	−5.4	−3.0

Z Only the fold changes greater (+ or up-regulation) or lower (− or down-regulation) than 2-fold are presented.

1. Apoptosis-related genes: Pz-DHA ester reduced the expression of pro-survival gene BCL2 (−2.8) whereas no significant change was shown on sorafenib treatment. Transcriptional activation of NF-κB1 was reduced by Pz-DHA ester (−4.3) or sorafenib (−2.5) treatment.

2. Growth factor receptors and its downstream signalling molecules: Pz-DHA ester reduced the expression of growth factors and receptor genes including IGF1R (−4.2), IGF2 (−2.3), PDGFRB (−16.8), EGFR (−8.9), ERBB2 (−5.7), ERBB3 (−6.1), KDR (−2.8), PDGFRA (−2.8). Pz-DHA esters inhibited PI3K/AKT/mTOR pathway with reduced expression of AKT1 (−6.1) and AKT2 (−2.8) and its effector GRB2 (−8.6). In parallel, mTOR (−3.6), PIK3C2A (−2.2), IRF5 (−2.3) and TP53 (−7.4) were also down regulated in Pz-DHA treated cells. Inactivation of KRAS (−4.0) on treatment with Pz-DHA ester demonstrated the significant down regulated expression of Ras/MAPK pathway in HepG2 cells. IGFIR (−2.1), IGF2 (−2.8), mTOR (−2.1), PDGFRB (−2.5) and NRAS (−2.4) were down regulated in sorafenib treated HepG2 cells.

3. Cell Cycle: Pz-DHA ester down regulated CDK2 (−2.0) CDK9 (−2.1), telomerase TERT (−5.6), Topoisomerases TOP2A (−2.7) and TOP2B (−2.3) and PARP1 (−5.4). Treatment with sorafenib reduced the expression of MDM4 (−2.3), TOP2B (−2.2) and PARP1 (−3.0) in HepG2 cells.

4. Protein kinases: Overexpression of protein kinases plays a major role in development and progression of HCC. AURKB (−2.0), PLK1 (−2.9), PRKCA (−8.1), PRKCD (−2.1) and PRKCE (−5.6) were down regulated on Pz-DHA ester treatment.

5. Histone deacetylases: HDAC1 (−2.6), HDAC11 (−20.1), HDAC4 (−4.1), HDAC6 (−5.4) and HDAC7 (−6.0) were down regulated by Pz-DHA ester.

The gene significantly up regulated in expression on Pz-DHA ester and sorafenib treatment is FLT1 (16.5).

## Discussion

The present work demonstrated the cytotoxicity and selectivity of novel fatty acid esters of phloridzin towards three human cancer cells, hepatocellular carcinoma, breast cancer and acute monocytes leukemia as compared to normal hepatocytes. The cytotoxic effect was drawn on the basis of lower EC_50_ values (<40 µM) and higher SI values (>3) that were obtained for the fatty acid esters of phloridzin. Based on literature to date [16] and present study, phloridzin have no inhibitory effect on cancer cells where as its aglycone, phloretin demonstrated anticancer property. This is agreeable with previous studies that phloretin augmented TRAIL-induced apoptosis and cytotoxicity in prostate cancer cells [15]. To our knowledge, this is the first study that has examined and demonstrated that fatty acid esters of phloridzin induce apoptosis in cancer cells. Fatty acid esters of phloridzin were synthesized in our laboratory by regioselective enzymatic acylation of phloridzin with six different long chain saturated, mono- and poly-unsaturated fatty acids. This study revealed that fatty acid esters of phloridzin were distinctly more potent inhibitors of the growth of solid tumors such as HCC, breast adenocarcinoma, and leukemia than their corresponding parent molecules, phloridzin and the six free fatty acids. Our results are in accordance with previous observations that chemically synthesized polyphenol, acetyl resveratrol analogues can inhibit growth of DU-145 human prostate cancer cells higher than that of resveratrol [23].

The antiproliferative effect suggests that the esterification of 6′-OH of glucose moiety of phloridzin with acyl side chains changed the bioactivity of phloridzin. This conformational change could lead to important modifications in the lipophilicity of the phloridzin molecule that modifies cellular permeability, uptake and/or interactions with membrane bound receptors. Synthetic derivatives of phloridzin formed by modification of sugar hydroxyl groups by esterification were shown to increase lipophilicity and antioxidant properties [24]. Furthermore, other studies have showed that the acetylation can enhance biological activities and bioavailability of epigallocatechin-3-gallate [25]. To demonstrate that acylation increases the bioavailability of fatty acid esters of phloridzin, in vivo studies need to be conducted to understand the stability and half-life of the novel esters in the intestine and plasma. Traditionally, medical oncologists have favoured intravenous drug therapy to treat most cancer patients. In an effort to overcome the obstacle of bioavailability, intravenous delivery of fatty acid esters of phloridzin is a major consideration that possibly yields greater bioavailability and therapeutic results than that of oral administration.

Our in vitro results demonstrated that fatty acid esters of phloridzin inhibit the growth of tumorigenic liver cells through the induction of apoptosis which are marked by induction of G_0_/G_1_ arrest, activation of caspase 3 that resulted in subsequent DNA fragmentation. Fatty acid esters of phloridzin treatment led to a loss of Δψm, suggesting that these compounds induce apoptosis, at least in part, through the mitochondrial pathway. Evidence can be found in literature on proapoptotic properties of synthetic analogues of polyphenols on cancer cells [26]. Chemotherapeutic agents that supress the proliferation of malignant cells by inducing apoptosis postulate a mechanism of their chemopreventive action [27]. Sorafenib has been reported to induce apoptosis and tumour necrosis in HCC [28]. Based upon previous studies, inhibition of topo IIα is a good target for anticancer drug in HCC [29], [30]. After topo IIα inhibition, apoptosis is the most efficient death pathway in human tumorigenic cells [31], [32]. In a cell-free screening experiment in this study, fatty acid esters of phloridzin, sorafenib, phloretin or phloridzin stopped the formation of cleavage complex and acted as catalytic inhibitor of topo IIα. Moreover, TOP2A transcriptional inhibition on Pz-DHA treatment correlates positively with catalytic inhibition of topo IIα in in vitro study. Phloretin was reported to inhibit DNA topo IIα unknotting activity and act as catalytic inhibitor of topo IIα enzyme [33].

Among the tested six novel esters, Pz-DHA ester had the greatest potential and efficacy as a chemotherapeutic agent. Thus, we have characterized the molecular mechanism and efficacy of Pz-DHA ester as a therapeutic for the treatment of HCC. Targeting with inhibitors directed at receptors and its different downstream signaling pathways is proven to be a more effective approach in anticancer therapy [34]. We found that programmed cell death results in part from the differential regulation of distinct signalling pathways in HepG2 cells evoked by the Pz-DHA ester. It is well known that growth factor receptors particularly epidermal growth factor receptor (EGFR) and insulin-like growth factor/IGF-1 receptor and their downstream signalling pathways activation is a key for diverse cellular processes such as cell proliferation, antiapoptosis and invasive behavior of tumor cells [35]. Activation of EGFR family induces a cascade of downstream signaling through PI3K/AKT/mTOR and mitogen-activated protein kinase (MAPK) resulting in cellular proliferation and differentiation [36]. Indeed, the fact that Pz-DHA ester treatment caused down regulation of the expression levels of EGFR, ERBB2, ERBB3, IGF and IGF-1R and its downstream signalling members PI3KC2A, AKT1, AKT2 and mTOR expression that had attributed to increased apoptotic cell death via caspase 3 signaling [36], [37]. Down-regulation of Grb2 adapter and Ras/Raf/MEK/ERK pathway by Pz-DHA ester contribute to the profound effect on proliferative and apoptotic pathways in HepG2 cells [38], [39]. In addition, transcription activity of nuclear factor kappa B (NF-κB) is known to be essential in cell proliferation and apoptosis via gene regulation. We found that Pz-DHA ester possessing the ability to suppress the expression of Protein Kinase C that down regulated NF-κB, PI3K/Akt/mTOR as well as MAPK pathway. In addition, our findings indicate that following Pz-DHA ester treatment down regulation of numerous gene expression via, cyclin-dependent kinases (CDK) such as CDK2 and CDK9 that contributes to cell cycle arrest in G_0_G_1_ phase that block entrance to other phase or mitosis in HepG2 cells [40]. The present study uncovered that Pz-DHA ester is very effective in inhibiting cancer associated enzyme telomerase along with cell cycle machinery. Studies reported that inhibition of telomerase by polyphenols might provide a plausible explanation for up-regulation of procaspase expressions [41] and the antiproliferative effects of dietary polyphenols [42].

Up-regulation of antiapoptotic proteins is one of the mechanisms employed by cancer cells to evade apoptosis [43]. Targeting the anti-apoptotic Bcl-2 family of proteins can improve apoptosis and thus overcome drug resistance to cancer chemotherapy [44]. In this study, we demonstrated that Bcl-2 down-regulation by Pz-DHA ester mediates mitochondrial membrane damage and apoptosis. It is notable that Bcl-2 protein regulates NF-κB pathway activation that increases the antiapoptotic threshold of cells by suppressing the initiation of caspase-8 activation [45]. Based upon these results, down-regulation of NF-κB and decreased expression of Bcl-2 on Pz-DHA ester treatment induces intrinsic apoptosis and facilitate elimination of transformed cells during hepatocellular carcinogenesis.

Epigenetic deregulations, in particular histone acetylation, play important roles in the pathogenesis and progression of HCC. Pz-DHA ester inhibited the best characterized and probably biologically most relevant histone deacetylases which are commonly dysregulated in cancer [46]. Several polyphenols including EGCG and resveratrol, are known to possess potent HDAC inhibitory activity [47]. HepG2 cells express a wild-type p53 protein [48] and p53 protein over-expression in tumors with wild-type gene has been found in several other studies [48]. It is worthy to note that Pz-DHA ester down-regulated TP53 is unexpected in an antineoplastic treatment. This may be reflective of the complex cellular response to the various signalling pathways by the ester.

In conclusion, this study demonstrated that the chemotherapeutic potential of six saturated, mono- and poly-unsaturated fatty acid esters of phloridzin. In addition to the substantial inhibitory effect against liver cancer cells, Pz-DHA ester had relatively low toxicity towards normal cells. Low toxicity was evident in normal human and rat hepatocytes treated with Pz-DHA ester compared with standard drug sorafenib. Pz-DHA ester also has potential impact on multimolecular targets and pathways that led to apoptosis compared to sorafenib. The HepG2 cell growth inhibition mechanism of these novel compounds is related to inhibition of topo IIα and triggered DNA damage, cell cycle arrest and apoptosis. DHA ester of phloridzin has the greatest potential and efficacy as a chemotherapeutic agent. The anti-proliferative properties of the ester seem to be through a mechanism that down regulate key signalling pathways including PI3K/AKT/mTOR. However, additional studies on Western blot analysis to confirm RT-PCR findings, caspase pathways, expression profiling and in vivo experiments are required to confirm the anti-cancer activity of these novel fatty acid esters of phloridzin.
